# The Effect of Long-Lasting Swimming on Rats Skeletal Muscles Energy Metabolism after Nine Days of Dexamethasone Treatment

**DOI:** 10.3390/ijms23020748

**Published:** 2022-01-11

**Authors:** Damian Jozef Flis, Emilia Gabriela Bialobrodzka, Ewa Aleksandra Rodziewicz-Flis, Zbigniew Jost, Andzelika Borkowska, Wieslaw Ziolkowski, Jan Jacek Kaczor

**Affiliations:** 1 Department of Pharmaceutical Pathophysiology, Faculty of Pharmacy, Medical University of Gdansk, Dębinki 7 Street, 80-211 Gdansk, Poland; 2 Poznan University of Physical Education, Królowej Jadwigi 27/39 Street, 61-871 Poznan, Poland; czyrko@awf.poznan.pl; 3 Department of Basic Physiotherapy, Gdansk University of Physical Education and Sport, K. Gorkiego 1 Street, 80-336 Gdansk, Poland; ewa.rodziewicz@awf.gda.pl; 4 Department of Biochemistry, Gdansk University of Physical Education and Sport, K. Gorkiego 1 Street, 80-336 Gdansk, Poland; zbigniew.jost@awf.gda.pl; 5 Department of Bioenergetics and Physiology of Exercise, Faculty of Health Sciences, Medical University of Gdansk, Dębinki 1 Street, 80-211 Gdansk, Poland; andzelika.borkowska@gumed.edu.pl; 6 Department of Rehabilitation Medicine, Faculty of Health Sciences, Medical University of Gdansk, Al. Zwycięstwa 30, 80-219 Gdansk, Poland; wieslaw.ziolkowski@gumed.edu.pl; 7 Department of Animal and Human Physiology, University of Gdansk, J. Bazynskiego 8 Street, 80-308 Gdansk, Poland; jan.kaczor@ug.edu.pl

**Keywords:** dexamethasone, endurance exercise, lipid metabolism, glucose metabolism, skeletal muscle

## Abstract

This study investigates the effect of Dexamethasone (Dex) treatment on blood and skeletal muscle metabolites level and skeletal muscle activity of enzymes related to energy metabolism after long-duration swimming. To evaluate whether Dex treatment, swimming, and combining these factors act on analyzed data, rats were randomly divided into four groups: saline treatment non-exercise and exercise and Dex treatment non-exercised and exercised. Animals in both exercised groups underwent long-lasting swimming. The concentration of lipids metabolites, glucose, and lactate were measured in skeletal muscles and blood according to standard colorimetric and fluorimetric methods. Also, activities of enzymes related to aerobic and anaerobic metabolism were measured in skeletal muscles. The results indicated that Dex treatment induced body mass loss and increased lipid metabolites in the rats’ blood but did not alter these changes in skeletal muscles. Interestingly, prolonged swimming applied after 9 days of Dex treatment significantly intensified changes induced by Dex; however, there was no difference in skeletal muscle enzymatic activities. This study shows for the first time the cumulative effect of exercise and Dex on selected elements of lipid metabolism, which seems to be essential for the patient’s health due to the common use of glucocorticoids like Dex.

## 1. Introduction

Glucocorticoids (GCs) have been used for more than 60 years for significant anti-inflammatory and immunosuppressive effects. Due to the broad spectrum of treatment options, the GCs are used in several acute and chronic disorders. In 1999, almost 1% of the United Kingdom population had prescribed GCs for long or short-term use [1]. Nevertheless, over the next 20 years, prescription of GCs across the whole world rose. Analysis of data from France patients indicated that from 2007 to 2014, the frequency of GC uses rose from 14 to 17% [2]. In the USA, administration of GC is even higher; up to 21% of American adults were given prescriptions for short-term use of oral corticosteroids during three years (2012–2014) [3].

One of the synthetic GCs used clinically is Dexamethasone (Dex). Dex is administrative to treat inflammatory and immune-mediated diseases, such as arthritis [4], allergic rhinitis [5], and asthma [6] and as part of various chemotherapy protocols [7].

Despite Dex’s broad range of indications, the long-term use of this GC is generally avoided because of the increased risk of adverse events in different tissues and organs [8]. Dex treatments may lead to disturbance in glucose metabolism and the development of diabetes [9]. Other adverse events of Dex administration are skeletal muscle atrophy and accelerated weight loss [10,11]. These negative changes are related to the Dex effect on FoxO-mediated ubiquitin-proteasome overactivity and impairment of IGF-1-mediated signaling [12]. Another coexisting mechanism of muscle atrophy induced by Dex is the upregulation of myostatin gene expression, which is also related to the swelling or vacuolization of mitochondria [13]. Moreover, Dex inducing skeletal muscle atrophy also results in other mitochondrial dysfunction.

After Dex treatment, Liu and coworkers observed loss of mitochondria content, compromised mitochondrial respiration, and disorders in mitochondrial dynamics [14]. These mitochondrial impairments may affect cellular processes as apoptosis, Ca^2+^ homeostasis, or energy production. Disturbances in energy production after Dex treatment are also confirmed by the metabolite levels in skeletal muscle [15]. After long-term Dex treatment, the observed changes are: slower nightly energy expenditure and accelerated fatty acid oxidation [16]. Interestingly, even 5 days of high-dose Dex treatment induced a significant increase in liver mass, an increase in liver mitochondrial non-phosphorylative O_2_ consumption rate, and a decrease in the thermodynamic coupling of oxidative phosphorylation in liver respiratory pathways. However, this treatment has no effect on energy metabolism in the gastrocnemius muscle [17]. Koski and coworkers presented the different effects of Dex treatment on skeletal muscles. They show decreased substrate oxidation by skeletal muscles of rats treated with Dex. The decrease appeared to be proportional to the content of white glycolytic fibers in the muscle [18]. The Weber group demonstrates the opposite effect of Dex treatment. Their results show that Dex stimulates mitochondrial biogenesis and that this phenomenon is relatively specific for skeletal muscle [19].

Despite these differences in energy metabolism after Dex treatment, skeletal muscle atrophy and changes in mitochondria functions occur as adverse events. Therefore, the potential protective therapies against muscle wasting are focused on improving mitochondria quality and content. One of the agents, which induced mitochondrial biogenesis via the AMPK–PGC-1α pathway is resveratrol. This polyphenol has been reported to be efficient in preventing muscle atrophy in several models as hypodynamia and hypokinesia [20], Dex treatment [21], non-alcoholic steatohepatitis [22], and sarcopenic obesity [23]. The previously administered physical training also prevented the muscle atrophy caused by Dex treatment.

Studies indicate that both high and low-intensity resistance training and endurance training prevent Dex-induced muscle atrophy. Eight weeks of resistance training ameliorate atrophy induced by Dex treatment via increased mTOR and reduced Atrogin-1 and MuRF-1 protein content [24,25]. In addition, endurance training also attenuates hyperglycemia and may prevent insulin resistance and muscular glycogen loss [26]. Despite the protective effects of physical training on skeletal muscle atrophy induced by Dex, there is no data relating to the effects of Dex on skeletal muscle metabolism under long-term endurance exercise conditions. Therefore, this study investigates the effect of long-time swimming on blood and skeletal muscle metabolites concentration and skeletal muscle activity of enzymes related to energy metabolism after Dex treatment.

## 2. Results

### 2.1. The Effect of Dex Treatment and Exercise on Rats Body Mass

**Figure 1:**
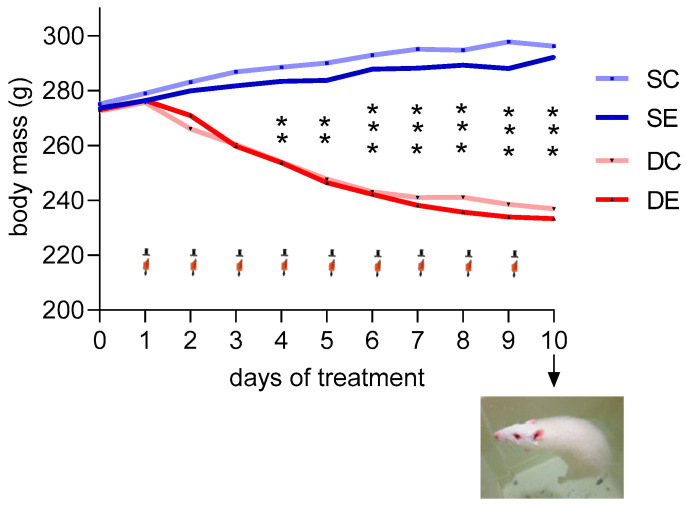
The effects of Dex treatment, swimming, and a combination of these factors on body mass during ten days observation. Body mass was measured daily for the entire experiment in saline-treated, non-exercised (SC), saline-treated, exercised (SE), Dex-treated, non-exercised (DC) and Dex-treated, exercised (DE) groups of rats. 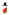
—Dex or saline injection, on the 10th day of the experiment, rats from exercised groups underwent swimming. ** *p* < 0.01, and *** *p* < 0.001 between saline (SC + SE) and Dex (DC + DE) treated rats. The data are presented as the means (*n* = 10 in each group).

Dex treatment significantly reduced body mass in both swimming and non-exercised animals compared to saline-treated rats. This change is time-dependent; the significant reduction was observed after 3 days of treatment (286.89 ± 4.32, 281.70 ± 7.37, 260.30 ± 10.91, and 259.70 ± 5.01 g in saline-treated, non-exercised (SC), saline-treated, exercised (SE), Dex-treated, non-exercised (DC) and Dex-treated, exercised (DE) groups, respectively) and the difference rose in the following days and the nine-day riches around 60 g (296.20 ± 4.68, 292.20 ± 6.98, 236.90 ± 9.08, and 233.30 ± 5.34 g in SC, SE, DC and DE groups, respectively; Figure 1).

### 2.2. Effects of Dex Treatment and Exercise on Liver and Muscle Damage Markers and Stress Response Elements

Neither nine days of Dex treatment and three hours of swimming, nor a combination of these conditions resulted in a change in blood creatine kinase (CK) and alanine aminotransferase (ALT) activities and cortisol (Cort) concentration. However, there was a trend towards increasing CK activity in the rats’ blood after Dex administration (*p* = 0.1). There was also a tendency to lower the Cort concentration in response to Dex treatment (*p* = 0.062, DC vs. SC groups) and a slight, insignificant increase in both exercised groups compared to non-exercise animals. Dex treatment also resulted in lowered plasma adrenaline (Adr) concentration compared to the SC group. There was also a slight, insignificant decrease in Adr concentration after swimming in both SE and DE groups compared to SC and DC groups (Table 1).

### 2.3. Effects of Dex Treatment on Glucose and Lactate Level under Exercise Condition

**Figure 2:**
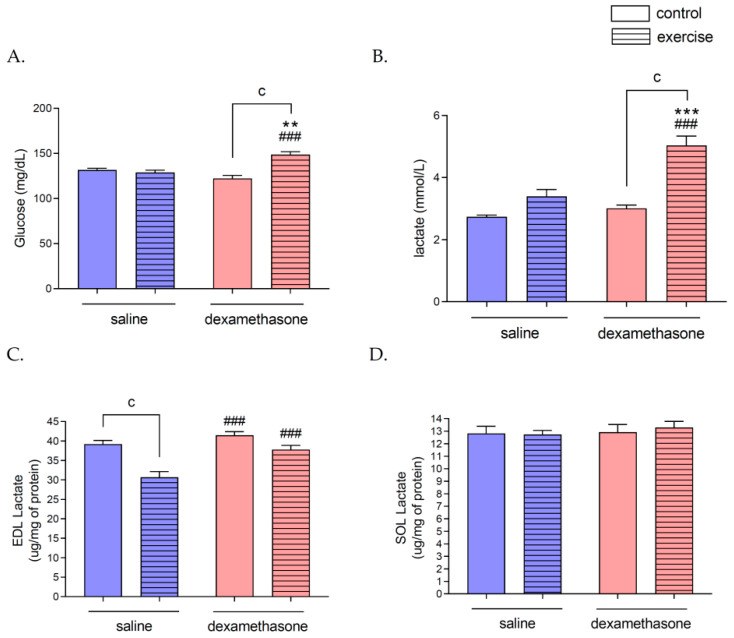
The effects of Dex treatment, swimming, and a combination of these factors on glucose and lactate concentrations in rat plasma and skeletal muscles. Blood Glucose (Glu) (**A**), Lactate (LA) (**B**), were measured in rats’ plasma. Soleus LA (**D**) and EDL LA (**C**) were measured in deproteinized muscle samples. There were significant differences between the groups: cp < 0.001 between the indicated groups; ^###^ *p* < 0.001 between the indicated group and saline exercised group; ** *p* < 0.01, and *** *p* < 0.001 between the indicated group and saline control group. The data are presented as the means ± SEM (*n* = 10 in each group).

In rats previously treated with Dex, swimming resulted in higher plasma Glu (148.50 ± 3.48 mg/dL) and LA (5.03 ± 0.31 mmol/L) than DC (*p* = 0.0002 for Glu and LA) and SE group (*p* = 0.0004 for Glu, *p* = 0.0002 for LA; Figure 2A,B). In the DE group, there were no changes in soleus (SOL) (13.29 ± 0.49 μg/mg of protein; Figure 2D) and extensor digitorum longus (EDL) (37.78 ± 1.08 μg/mg of protein) LA concentration as compared to the DC group, but the lactate level in EDL was higher in the DE group than in the SE group (*p* = 0.0006; Figure 2C).

### 2.4. Effects of Dex Treatment on Glucose and Lactate Concentrations in Rat Plasma and Skeletal Muscles

Nine days of Dex treatment did not change the level of glucose (Glu) (122.19 ± 3.34 mg/dL) and lactate (LA) (3.01 ± 0.11 mmol/L) in the blood of animals compared to the SC group (131.56 ± 2.08 mg/dL and 2.74 ± 0.06 mmol/L for Glu and LA, respectively; Figure 2A,B). There were also no changes in LA concentration in SOL (12.93 ± 0.61 μg/mg of protein) and EDL muscles (41.45 ± 0.93 μg/mg of protein) as compared to the SC group (12.81 ± 0.58 and 39.16 ± 0.97 μg/mg of protein in SOL and EDL, respectively; Figure 2C,D). 

### 2.5. Effects of Swimming on Glucose and Lactate Levels in Rat Plasma and Skeletal Muscles

Three hours of swimming did not change the concentration of Glu (128.83 ± 2.59 mg/dL) and LA (3.39 ± 0.22 mmol/L) in the plasma of rats compared to the SC group (Figure 2A,B). After this type of exercise, there was no change in the level of lactate in the SOL muscle (12.74 ± 0.32 μg/mg of protein; Figure 2D), but we observed a significant decrease in LA in the EDL muscle (30.64 ± 1.45 μg/mg of protein) compared to the SC group (*p* = 0.0002; Figure 2C). 

### 2.6. Effects of Dex Treatment on Lipid Metabolites Level in Rat Plasma and Skeletal Muscles under Exercise Condition

**Figure 3:**
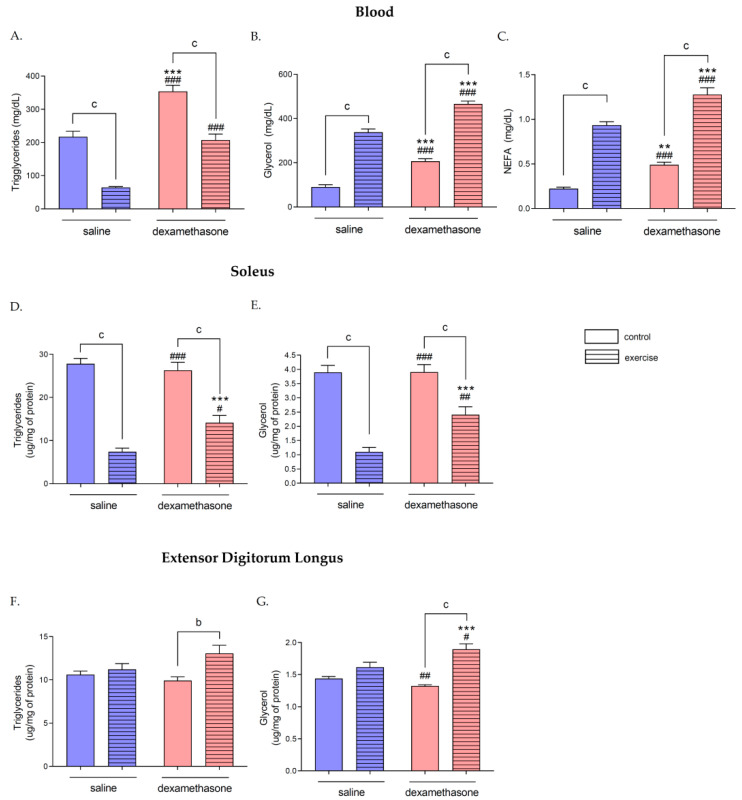
The effects of Dex treatment, swimming, and a combination of these factors on lipid metabolites level in rat plasma and skeletal muscles. Blood Triglycerides (Trigs) (**A**), Glycerol (Gly) (**B**), and Non-Esterified Fatty Acids (NEFA) (**C**) were measured in rats’ plasma. Soleus Trigs (**D**) and Gly (**E**), as well as EDL Trigs (**F**) and Gly (**G**), were measured in deproteinized muscle samples. There were significant differences between the groups: ^b^ *p* < 0.01, ^c^ *p* < 0.001 between the indicated groups; ^#^ *p* < 0.05, ^##^ *p* < 0.01, and ^###^ *p* < 0.001 between the indicated group and saline exercised group; ** *p* < 0.01, and *** *p* < 0.001 between the indicated and saline control groups. The data are presented as the means ± SEM (*n* = 10 in each group).

Swimming in rats previously treated with Dex (DE group) resulted in the same tendency of change in plasma metabolites concentration compared to the DC group as in saline treatment animals; however, the concentration of Trigs (207.13 ± 18.35 mg/dL), Gly (465.66 ± 13.02 mg/dL), and NEFA (1.28 ± 0.08 mg/dL) were higher in DE than in the SE group *p* = 0.0002 for Trigs and Gly, *p* = 0.002 for NEFA; Figure 3A–C). A similar observation was noticed in SOL muscle, where Trigs (14.10 ± 1.73 μg/mg of protein) and Gly (2.41 ± 0.28 μg/mg of protein) levels decreased after swimming compared to the DC group (*p* = 0.0002 for Trigs, *p* = 0.0007 for Gly), but was significantly higher than in SE (*p* = 0.02 for Trigs, *p* = 0.03 for Gly; Figure 3D,E). Interestingly, swimming after Dex treatment increased both Trigs (13.06 ± 0.95 μg/mg of protein) and Gly (1.89 ± 0.09 μg/mg of protein) concentration in EDL muscle compared to the DC group (9.91 ± 0.43 and 1.32 ± 0.02 μg/mg of protein for Trigs and Gly, respectively; *p* = 0.009 for Trigs, *p* = 0.0002 for Gly; Figure 3F,G).

### 2.7. Effects of Dex Treatment on Lipid Metabolites Level in Rat Plasma and Skeletal Muscles

Nine days of Dex treatment induced accumulation of triglycerides (Trigs) (353.73 ± 18.82 mg/dL), glycerol (Gly) (206.60 ± 11.44 mg/dL), and non-esterified fatty acids (NEFA) (0.49 ± 0.03 mg/dL) in rats plasma compared to SC group (217.05 ± 16.63, 90.45 ± 10.52 and 0.22 ± 0.02 mg/dL for Trigs, Gly and NEFA concentration, respectively, *p* = 0.0002 for Trigs and Gly and *p* = 0.002 for NEFA; Figure 3A–C). However, this treatment did not induce any changes in Trigs and Gly concentrations in SOL and EDL skeletal muscles (Figure 3D–G). 

### 2.8. Effects of Swimming on Lipid Metabolites Level in Rat Plasma and Skeletal Muscles 

Three hours of swimming significantly lowered Trigs levels in plasma (64.66 ± 2.59 in SE, and 217.05 ± 16.63 mg/dL in SC group, *p* = 0.0002; Figure 3A) and SOL muscle (7.37 ± 0.87 in SE group, and 27.78 ± 1.25 μg/mg of protein in SC group, *p* = 0.0002; Figure 3D) and did not affect the Trigs level in EDL muscle in comparison to SC groups (Figure 3F). These changes were related to an increase in plasma Gly (337.52 ± 15.16 in SE group, and 90.45 ± 10.52 mg/dL in SC group, *p* = 0.0002; Figure 3B) and NEFA (0.93 ± 0.04 in SE group, and 0.22 ± 0.02 mg/dL in SC group, *p* = 0.0002; Figure 3C) concentrations. Swimming also decreased the Gly level in SOL muscle (1.10 ± 0.16 in the SE group, and 3.89 ± 0.25 μg/mg of protein in the SC group, *p* = 0.0002; Figure 3E) but did not affect EDL Gly level (Figure 3G). 

### 2.9. Effects of Dex Treatment and Exercise on Enzymes Related to Aerobic and Anaerobic Metabolism and PGC-1α Level in Skeletal Muscles 

**Figure 4:**
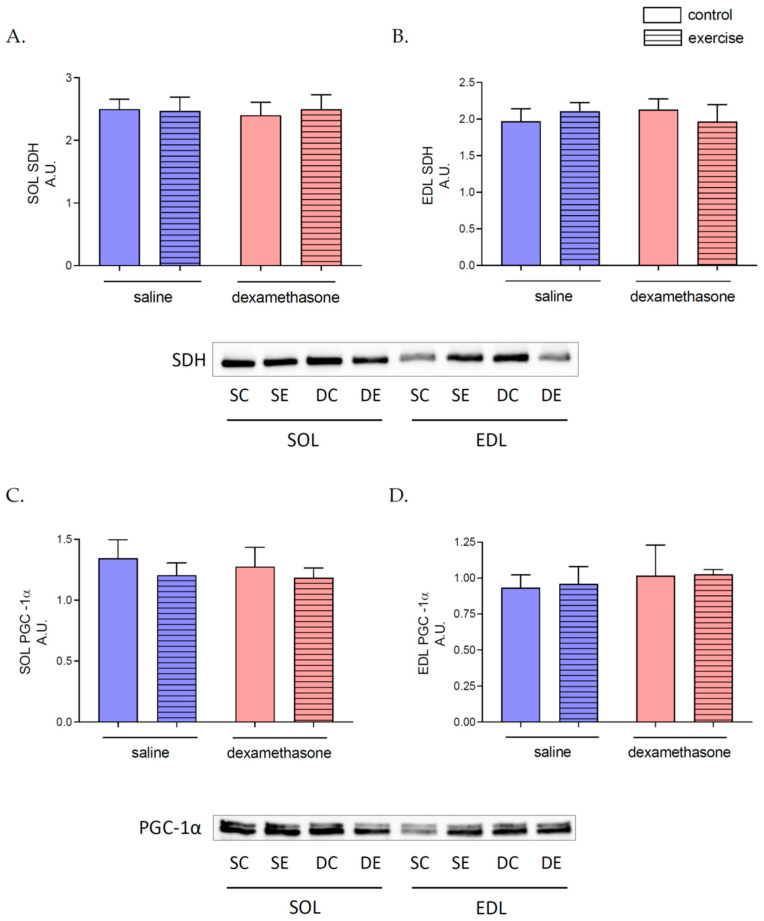
The effects of Dex treatment, swimming, and a combination of these factors on succinate dehydrogenase and peroxisome proliferator-activated receptor gamma coactivator 1-alpha levels in skeletal muscles. SOL succinate dehydrogenase (SDH) (**A**) and peroxisome proliferator-activated receptor gamma coactivator 1-alpha (PGC-1α) (**C**) levels, as well as EDL SDH (**B**) and PGC-1α (**D**), were measured in skeletal muscles lysates in saline-treated, non-exercised (SC), saline-treated, exercised (SE), Dex-treated, non-exercised (DC) and Dex-treated, exercised (DE) groups of rats. Changes in protein levels were assessed by densitometry of the immunoreactive bands and normalized to the total amount of protein in the samples transferred onto the membrane (Figure S1). The data are presented as the means ± SEM (*n* = 6 in each group).

Neither nine days of Dex treatment and three hours of swimming nor a combination of these conditions resulted in any changes in activities of enzymes related to aerobic (citrate synthase (CS) and cytochrome c oxidase (COX)) and anaerobic (lactate dehydrogenase (LDH)) metabolism in both SOL and EDL skeletal muscles (Table 2) as well as the level of succinate dehydrogenase (Figure 4A,B) and PGC-1α (Figure 4C,D) in these muscles. 

### 2.10. Effects of Dex Treatment and Exercise on Mitochondrial Function

Neither nine days of Dex treatment and three hours of swimming, nor a combination of these conditions resulted in any changes in mitochondrial respiration rates: non-phosphorylating LEAK respiration, OXPHOS capacity, NADH and succinate-linked OXPHOS capacity or mitochondrial coupling efficiency (Table 3).

## 3. Discussion

In this study, we investigate, for the first time, the post-exercise metabolites levels, liver and muscle damage markers, and stress response elements under the influence of Dex treatment. The main finding of this work is that the nine days Dex treatment modifies the long-term swimming-induced metabolic response in rats seen in both blood and skeletal muscles. Furthermore, Dex-treatment induces lowering of rats body mass which is accompanied by changes in blood lipid metabolites. It is worth emphasizing that these changes are not related to rats’ skeletal muscles or liver damage. Interestingly, prolonged swimming induced a different response in the levels of the metabolites mentioned above, especially in the white, fast-twitched EDL muscle. 

One of the most common adverse events after Dex treatment is lowering body mass, which was also observed in our study. Interestingly, even topical ocular Dex treatment induced body mass loss, liver damage, and blood cholesterol alteration in rats [27]. Therefore, to evaluate whether intraperitoneal injection of Dex, swimming, and combining these factors induce muscle or liver damage or influence stress response elements, we measured CK, ALT activities and Cort, and Adr concentrations in the rats’ plasma. As mentioned before, we observed changes in neither skeletal muscle nor liver damage markers, but we observed a tendency to increase the plasma CK activity after Dex treatment. This observation is in line with previously published papers, because, on the one hand, Noh and coworkers documented that 5 days of Dex administration resulted in a 3-fold increase of CK serum level in rats [28]. However, on the other hand, other authors showed that Dex administration might even decrease the level of circulating CK levels, which may be related to protein synthesis inhibition, cellular membrane stabilization, anti-inflammatory, and catabolic effects on myofibers [29,30]. Changes in the level of blood ALT are Dex dose-dependent. In higher than clinical dose levels of Dex treatment, ALT increased by almost 10-fold after 4 days of administration [31], but the lower dosage of Dex did not alter blood ALT activity even after 12 days [32]. To evaluate whether Dex treatment or swimming acts as a stressor for animals, we measured Cort and Adr concentrations in rats’ plasma. In Dex treated rats group, we observed a tendency to lower the blood Cort level. This change is related to the influence of Dex on the HPA axis. After administration of Dex, authors observed lowering of Cort blood level both in animal and human studies [33,34]. However, a slight, insignificant increase in Cort level was observed after swimming in both groups, which may be considered as a factor of stress related to swimming. After Dex treatment, Adr level drops, which may be related to Dex’s inhibitory action on releasing this hormone [35]. No changes in Adr level were observed after exercise in both groups.

Because Dex treatment may impair glucose metabolism and homeostasis, we evaluate whether tested factors will influence blood glucose and lactate as well as skeletal muscles lactate levels. 

Glucose is an essential fuel for contracting skeletal muscle during prolonged, strenuous exercise. Skeletal muscle glucose uptake is determined primarily by exercise intensity, duration, and glucose supply, as well as circulating hormones [36]. In low-intensity exercises, glucose in skeletal muscles is transformed into pyruvate and transported into mitochondria; however, when the intensity of exercise increases, it “switches on” the anaerobic possibility to metabolize the glucose. In this condition, lactate production and increased blood lactate concentration were observed [37]. Therefore, we measured blood glucose and lactate and SOL and EDL lactate concentrations to evaluate whether Dex treatment, swimming, and combining these factors influence skeletal muscle glucose metabolism. 

Neither three hours of swimming nor nine days of Dex treatment altered blood glucose levels. Nevertheless, long-lasting swimming in animals previously treated by Dex resulted in higher blood glucose concentration. These results align with previously published data, where authors indicated that Dex-treatment induces diabetes-related symptoms like hyperinsulinemia and hypertriglyceridemia even without changes in fasting glucose level [9]. Another action of Dex treatment on glucose metabolism is inhibition of skeletal muscle glucose uptake [38], which may explain the increased glucose level after exercise. 

There was also an increase in blood lactate level after swimming, but this was statistically significant only in the Dex treated group of animals. Therefore, we assume that the same additional load (3% of actual body weight of the animal) generates a different intensity of exercise in both groups of animals, and this response may be related to impaired metabolism caused by Dex treatment [39]. The blood level of lactate during exercise is related to the production of lactate by skeletal muscle and the possibility of its utilization. Our study indicated that neither Dex treatment, swimming, nor combining these factors influenced the lactate concentration in red, slow-twitched SOL muscle. In opposite in white, fast-twitched EDL muscle, there was a reduction in lactate concentration after 3 h of swimming, but, interestingly, this change was not found after Dex treatment, where lactate concentration was higher after exercise compared with the SE group.

Lactate production in muscle cells provides energy to continued exercise, but lactate is not an undesirable reaction product. This molecule is also a fuel energy source, gluconeogenic precursor, and signaling molecule [40]. One of the cellular signals induced by lactate is regulating skeletal muscle lipid metabolism [41]. Therefore, we investigate the effects of the conditions mentioned above also on lipid metabolism.

Usage of lipids as a primary energetic substrate is observed in the long-lasting, moderate-intensity endurance exercises. In these types of exercises, there is an increase in lipolysis in adipose tissue, as well as in the working muscles [42]. Our study found that after 3 h of swimming, there was an increase in plasma NEFA and glycerol level, which should be considered as metabolites related to increased adipose tissue lipolysis [43]. Higher circulating Gly level could also be related to skeletal muscle lipolysis and release of Gly from skeletal muscle to blood mainly observed in slow-twitched muscles under long-lasting swimming. Another factor that proves these changes is the lowering of SOL and blood concentration of Trigs, which are the storage form of fatty acids. Mentioned above changes did not occur in the fast-twitched EDL muscle, which should be related to the anaerobic–glycolytic nature of these types of muscle. 

Nine days of Dex treatment increased blood Trigs, Gly, and NEFA concentrations in non-exercised animals. These changes should be considered a result of the activation of lipolysis in adipose tissue by GCs [44], especially since the level of these metabolites in skeletal muscles remains unchanged. Also, reduction of animals mass after Dex treatment should be considered as additional proof for adipose tissue lipolysis. However, hypertriglyceridemia induced by Dex treatment is also related to early diabetes [9]. Interestingly, long-lasting swimming in animals previously treated with Dex resulted in similar changes in blood lipid metabolites as in the non-Dex treated animals; however, the concentration of these metabolites was higher than in the control group. Also, the level of Trigs and Gly in SOL muscle declined after swimming, but the concentration of these metabolites was higher than in non-Dex treatment swimming animals. The differences in the concentration of these metabolites after exercise should be considered as another result confirming higher swimming intensity in the Dex-treated group of rats.

Interestingly, swimming after Dex treatment results in the increase of both Trigs and Gly in fast-twitched EDL muscle. These changes between two types of skeletal muscles may result from lipolysis rate, which is higher in SOL muscle, and the possibility of muscle type to Gly release under lipolysis state [45]. Another coexisting explanation of Trigs accumulation in EDL after 3 h of swimming is lactate, which is produced mainly by muscles composed of fast-twitch types of fibers. The study of Sun et al. indicated that lactate is also a signaling molecule, which via inhibition of the cAMP-PKA pathway and activation of GPR81, may lead to intramuscular accumulation of Trigs [41].

Changes in the glucose and lipid metabolites led us to verify whether these changes are related to the impairment of aerobic and anaerobic enzymatic activities.

The production of energy by skeletal muscle is related to the activities and levels of specific enzymes. In cells, some enzymes represent anaerobic metabolism, i.e., citrate synthase (one of the Krebs cycle enzymes), cytochrome c oxidase (one of the electron transport chain enzymes), and also enzymes related to anaerobic metabolism like lactate dehydrogenase [46]. Our study showed no changes in any tested aerobic and anaerobic enzymatic activities and SDH levels after Dex treatment or long-lasting swimming in both types of skeletal muscles. These treatment types do not induce changes in skeletal muscle PGC-1α protein content, which stimulates mitochondrial biogenesis and promotes the remodeling of muscle tissue to a fiber-type composition that is metabolically more oxidative and less glycolytic [47]. What is more, there are no changes in skeletal muscle mitochondrial respirations states and OXPHOS coupling efficiency. In the scientific literature, there are controversies related to the influence of Dex and other GCs on skeletal muscle metabolism. In literature data, there are research: showing no effects of Dex treatment on skeletal muscle energy metabolism [17], decreased substrate oxidation by skeletal muscles [18], and even stimulation of mitochondrial biogenesis [19]. It seems that the skeletal muscle response to Dex treatment is related to many factors, like dosage, time of treatment, or even the method of administration. In research published by Mitsui et al., authors indicated that chronic GCs treatment leads to human skeletal muscle mitochondria dysfunction; however, they observed changes in complex I of electron transport chain activity, but not in the COX activities [48]. On the animal model, five days of Dex treatment lowered COX activity in skeletal muscle simultaneously, with no changes in CS activities [17]. Our research also confirms no effect of Dex treatment on LDH activities in both types of skeletal muscles [49]. 

**Figure 5:**
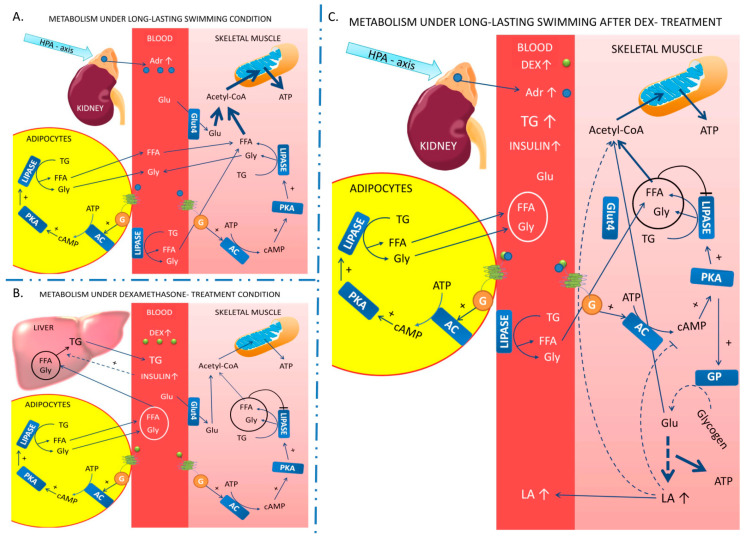
Potential mechanism(s) of action of swimming (**A**), Dex treatment (**B**), and a combination of these factors (**C**) on energy metabolism.

Based on [41,50,51,52] and our results, we propose a potential mechanism(s) of action of swimming (A), Dex treatment (B), and a combination of these factors (C) on energy metabolism (Figure 5).

(A) Like any other type of exercise, long-lasting swimming increases energy expenditure. Therefore, to resynthesize more ATP for working muscles, the organism activates the HPA axis to increase releasing of adrenalin from adrenal glands into the blood. Adrenalin acts on adipocytes and skeletal muscles via a β-adrenergic receptor (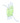), which stimulates (+) (via G protein, G) production of cAMP by adenylyl cyclase (AC) and following activates the Protein Kinase A (PKA). PKA activates enzymes related to the lysis of triglycerides (TG) and glycogen-lipase, and glycogen phosphorylase (GP). After activation, the rate of lipolysis rise in adipocytes and skeletal muscles, which is related to increasing the concentration of free fatty acids (FFA) and glycerol (Gly). These metabolites are transported from lipid cells to blood, and FFA enter to skeletal muscle. In skeletal muscle, FFA are transformed to acetyl-CoA and transported into mitochondria, where β-oxidation, Krebs cycle, and electron transport chain cooperate to resynthesis ATP. Our data suggest that long-lasting swimming supports the switch to fatty acid oxidation in skeletal muscle through activation in the HPA axis and turned on regulatory cascade rather than gross changes in mitochondrial volume or mitochondrial turnover.

(B) Dexamethasone (Dex), as a glucocorticoid, acts on adipocytes and skeletal muscles like adrenaline, which leads to the accumulation of FFA and Gly (metabolites in the circle) in the blood. In contrast to exercise conditions, where energy expenditure increases, FFA are accumulated and not activated in skeletal muscle cells, leading to feedback inhibition (Ͱ) of lipase activity. FFA from the blood, instead of to muscle, are transported into the liver, and in hyperinsulinemia state, TG are synthesized from FFA and Gly. Next, TG are released into the blood. 

(C) Exercise in animals previously treated with Dex results in different molecular responses, especially in white, fast-twitched EDL muscle. Because feedback lipase inhibition exist and the energy expenditure rises, the organism needs to resynthesize ATP from not only lipids sources, but also glycogen and glucose (Glu). Metabolism of Glu may be aerobic (mitochondrial) and anaerobic (mainly in fast-twitched muscles). Under these conditions, Glu can be transformed into pyruvate and, next, depending on the situation, in acetyl-CoA or lactic acid (LA). Under exercise conditions, LA may be used as an energy source (in slow-twitched muscles), accumulate inside the skeletal muscle, or be transported to the bloodstream. In skeletal muscle, accumulated LA may also play a role as a signaling molecule. In this situation, LA inhibits cAMP-PKA signaling and leads to the accumulation of TG in skeletal muscle. 

A major limitation of this study is the lack of skeletal muscle transcriptome analysis, which may show whether Dex treatment affects the mRNA level related to energy metabolism proteins. Further studies are required to evaluate whether the different periods of Dex treatment will induce other changes in exercise energy metabolism and if Dex treatment also changes the energy metabolism under different types of exercises. Also, in the following research related to Dex treatment, a glucose tolerance test should be done to verify if this treatment induced pre-diabetes or diabetes. 

## 4. Materials and Methods

### 4.1. Animals

All experimental procedures, which included minimizing the number of animals and their suffering, were reviewed and approved by the 3rd Local Ethical Committee for Experiments on Animals in Gdansk (decision number 51/2015, 14 December 2015). Guidelines for the handling use and ethical treatment of laboratory animals based on European Union Directive 2010/63/E.U. were followed in all experiments.

Male Wistar rats (four groups, *n* = 10 per group), weighing 250–300 g, were housed in an environmentally controlled room (23 ± 1 °C with a 12 h light-dark cycle) and received standard rat chow and water ad libitum. After acclimatization, rats were randomly assigned into the following groups: SC- saline treatment, sedentary group; SE- saline treatment, exercise group; DC–Dex treatment, sedentary group; DE–Dex treatment, exercise group. Animals were weighed every day, at the same time, to calculate the dose of Dex as well as exercise loading.

The animals were sacrificed by stunning and decapitation. After decapitation, blood was collected into a tube containing EDTA and centrifuged at 2000× *g* to obtain plasma. Next, plasma was kept at –80 °C until analysis. 20 uL of blood was also added to 180 uL of 0.4 M perchloric acid to deproteinized sample. After vortex, deproteinized blood was centrifuged at 14,000× *g* and stored for lactate analysis. Also, skeletal muscles (SOL and EDL) were rapidly removed and frozen in liquid nitrogen and kept at –80 °C until analysis. 

### 4.2. Intraperitoneal Injection of Dexamethasone or Saline

The SC and SE groups received saline (the same amount as dexamethasone groups), the DC and DE groups will receive 1 mg/kg of Dexamethasone (dissolved in saline) every other day by intraperitoneal injection for 9 days. 

### 4.3. Exercise Protocol

The rats were prepared for the experiments and exercise tests using the methods described previously [53]. Before the experiments, the animals in the SE and DE groups were acclimatized to reduce swimming stress. Each day during the preparatory procedure, the rats swam for 30 min in water at 35 °C. On the first day, the rats swam without any additional weight. On the second, third, and fourth days, the rats swam burdened with 1, 2, and 3% of their body weight, respectively. Additional weights, placed on the rope, were attached with plaster at the height of 3/4 of the distal part of the rat’s tail. On the fifth day, exercise testing was performed in the SE and DE groups of rats, which consisted of 3 h of prolonged swimming in 35 °C water burdened with 3% of their body weight. Immediately after completing their protocol, the rats were euthanized (as described in the “Animals” subsection). 

### 4.4. Preparation of Skeletal Muscle for Analysis

#### 4.4.1. Enzymatic Activities and Metabolites Levels

After unfrozen, skeletal muscle was weighted and immediately inserted into cold homogenization buffer (50 mM Potassium Phosphate, 1 mM EDTA, 0.5 mM DTT, 1.15% KCl, pH 7.4, supplemented with protease inhibitor cocktail). Next, the 5% homogenate was made in a hand glass homogenizer. After obtaining homogenate (around 30 stokes), the homogenate was separated into two tubes, one for enzymatic activities measurement (immediately frozen and kept at –80 °C until analysis), and the second for analysis of metabolites level. To measure levels of metabolites in skeletal muscles, the samples were deproteinized by mixing the same volume of homogenate and 0.8 M Perchloric acid. Next, the samples were centrifuged at 14,000× *g*, and the supernatant was collected and stored until analysis at –80 °C.

#### 4.4.2. Western Blotting

After unfrozen, skeletal muscle was weighted and immediately inserted into cold lysis buffer (RIPA buffer, supplemented with protease inhibitor cocktail). Next, the 10% lysate was made in a hand glass homogenizer. Next, the lysate was frozen at –80 °C and thawed at 30 °C, three times and re-homogenized. Finally, the material was centrifuged at 15,000× *g* for 10 min at 4 °C. The resulting supernatant was decanted and frozen at –80 °C for further analysis. 

### 4.5. Metabolites Level Measurement

#### 4.5.1. Blood Glucose Level

The glucose level was measured in plasma using the manufacturer’s instructions from RANDOX GLUC-PAP assay (cat. number GL 2623, Randox Laboratories Ltd., Crumlin, County Antrim, UK), against the standard curve, according to the manufacturer’s instructions.

#### 4.5.2. Blood and Skeletal Muscle Lactate Concentration

The lactate level was measured in deproteinized blood and skeletal muscle samples, according to [54]. Briefly, 10 uL of deproteinized SOL, EDL, and blood samples were mixed with 250 uL of cocktail (1.1 M hydrazine buffer, pH 9.0; 0.25 mM NAD and 1 U LDH/mL). Next, 96-well plate was incubated for 30 min in the dark at 25 °C. After incubation, the fluorescence was measured at Ex = 365 nm, Em = 410–460 nm by GloMax^®^-Multi Detection System (Promega Corporation, Madison, WI, USA).

#### 4.5.3. Blood and Skeletal Muscle Triglycerides Concentration

The glycerol level was measured in plasma and deproteinized muscle samples using a RANDOX Triglycerides assay (cat. number TR 210, Randox Laboratories Ltd., Crumlin, County Antrim, UK), against the standard curve, according to the manufacturer’s instructions.

#### 4.5.4. Blood and Skeletal Muscle Glycerol Concentration

The glycerol level was measured in plasma and deproteinized muscle samples using a RANDOX Glycerol assay (cat. number GY 105, Randox Laboratories Ltd., Crumlin, County Antrim, UK), against the standard curve, according to the manufacturer’s instructions.

#### 4.5.5. Blood Non-Esterified Fatty Acid Level

The NEFA level was measured in plasma using a RANDOX NEFA assay (cat. number FA 115, Randox Laboratories Ltd., Crumlin, County Antrim, UK) against the standard curve, according to the manufacturer’s instructions.

#### 4.5.6. Blood Alanine Aminotransferase Activity

The ALT activity was measured in plasma using a RANDOX ALT assay (cat. number AL 146, Randox Laboratories Ltd., Crumlin, County Antrim, UK) against the standard curve, according to the manufacturer’s instructions.

### 4.6. Blood Adrenaline Concentration

The adrenalin concentration was measured in plasma using a Demeditec Diagnostics Adrenaline ELISA (cat. number DEE6100, Demeditec Diagnostics GmbH, Kiel, Germany) to the manufacturer’s instructions.

### 4.7. Blood Cortisol Concentration

The cortisol concentration was measured in plasma using an Arbor Assays Cortisol enzyme immunoassay kit (cat. number K003-H1/H5 Arbor Assays, Ann Arbor, MI, USA), according to the manufacturer’s instructions.

### 4.8. Enzymatic Activities Measurements

All enzymes activities were measured spectrophotometrically (Cecil CE9200, Cecil Instruments Limited, Cambridge, UK) in muscles homogenates. 

#### 4.8.1. Citrate Synthase

The citrate synthase (CS) activity was measured at 37 °C in duplicate according to [55]. Briefly, 10 μL of homogenate (1:10, 5%) was incubated for 2 min in 970 μL of buffer (50 mM Tris-HCl, 1 mM EDTA, 0.01% Triton-X100, pH 7.8) supplemented with 10 μL of freshly made DTNB (10 mM) and 10 μL acetylCoA (50 mM). Next, 10 μL of freshly made oxaloacetic acid (10 mM) was added to initiate the reaction. The reactions were conducted in duplicate, and absorbance was read at 412 nm.

#### 4.8.2. Cytochrome Coxidase

The cytochrome c oxidase (COX) activity was measured at 37 °C, according to [56]. Briefly, 20 μL of homogenate (1:10, 5%) was incubated for 2 min in 960 μL of buffer (10 mM potassium phosphate buffer, 0.01% Triton-X100, pH 7.2). Next, 20 μL of reduced cytochrome c was added to initiate the reaction. The reactions were conducted in duplicate, and absorbance was read at 550 nm.

#### 4.8.3. Lactate Dehydrogenases

The lactate dehydrogenase (LDH) activity was measured at 30 °C according to [57]. The two concentrations of pyruvate (PYR) were used to determine the maximal activity of lactate dehydrogenase characteristics for subunit M4 (LDH 2.1) in the presence of 2.1 mM PYR and subunit H4 (LDH 0.3) in the presence of 0.3 mM PYR. 

Briefly, 20 μL of homogenate (1:10, 5%) for SOL or 10 μL of homogenate (1:10, 5%) for EDL was incubated for 2 min in buffer (50 mM potassium phosphate buffer, 1 mM EDTA, 0.01% Triton-X100, pH 7.4) supplemented with 10 μL of freshly made NADH (20 mM). Next, 10 μL (LDH 2.1) or 1.43 μL (LDH 0.3) of pyruvate was added to initiate the reaction. The reactions were conducted in duplicate, and absorbance was read at 340 nm.

### 4.9. Visualization of SDH and PGC-1α Protein Levels

Equal amounts of muscle lysates (50 μg of protein per sample) were separated on 4–20% SDS-polyacrylamide gradient gels and transferred onto a polyvinylidene difluoride membrane. The analysis procedure was performed according to [58]. The following antibodies were used: rabbit monoclonal IgG anti-SDH (cat. No. #11998, 1:1000; Cell Signaling, Beverly, MA, USA); rabbit polyclonal anti-PGC-1α (cat. no. sc-13067, 1:500; Santa Cruz Biotechnology, Dallas, TX, USA); anti-rabbit IgG–peroxidase conjugate (cat. no. A9169, 1:25,000; Sigma Aldrich, St. Louis, MI, USA). After incubation in primary (overnight at 4°C) and secondary antibodies (1 h at room temperature), immunoblots were detected and visualized using enhanced chemiluminescence reagents (Western Lightning Plus ECL, Perkin Elmer, Waltham, MA, USA). Changes in protein levels were assessed by densitometry of the immunoreactive bands and normalized to the total amount of protein in the samples transferred onto the membrane (Figure S1) [59]. Relative protein levels were analyzed and quantified using ChemiDoc image analysis system (Bio-Rad Laboratories, Inc., Hercules, CA, USA). The immunoblotting analyses were done for six randomly selected animals from each group.

### 4.10. Isolation of Quadriceps Mitochondria

The skeletal muscle mitochondria were isolated, as previously described by Fontaine et al. [60], with slight modifications [53]. The quadriceps muscle was dissected from the surrounding connective tissue, rapidly removed, trimmed clean of visible connective tissue, weighed, and placed in 10 mL of ice-cold mitochondrial isolation buffer A (mM: 100 KCl, 50 Tris base, 5 MgCl_2_, 5 EDTA, pH 7.4). Muscles were minced with scissors, incubated for 1 min with Nagarse protease (10 mL of isolation buffer per 1 g of tissue, supplemented with Nagarse (0.2 mg mL^−1^)). Next, the same volume of isolation buffer A was added to inhibit protease activity, and tissue was homogenized using a Teflon pestle homogenizer. The homogenate was then centrifuged at 700× *g* for 10 min. The supernatant was decanted and centrifuged at 10,000× *g* for 10 min. The pellet was resuspended in 40 mL of suspension buffer B (mM: 100 KCl, 50 Tris base, 1 MgCl_2_, 1 EDTA, 0.5% BSA, pH 7.4)) and centrifuged at 10,000× *g* for 10 min. This washing step was repeated two times. The final mitochondrial pellet was resuspended in buffer B without BSA (0.25 μL of buffer B per 1 g of muscle mass).

All steps were performed at 4 °C.

### 4.11. High-Resolution Respirometry and Mitochondrial Quality and Control 

Respiration was measured at 37 °C under constant stirring (750 rpm), which ensured a homogenous oxygen distribution in the medium, in a high-resolution respirometer using an Oxygraph-2k (O2k, Oroboros Instruments, Innsbruck, Austria), a modular system for high-resolution respirometry (HRR). Oxygen concentration (μM) and oxygen flux per mass [pmol O2∙s^−1^∙mg^–13^] were recorded in real-time while obtained data were evaluated using DatLab software (Oroboros Instruments, Innsbruck, Austria).

Briefly, mitochondrial respiration was measured in MiR05 (mitochondrial respiration medium containing mM: 0.5 EGTA, 3 MgCl2∙6H2O, 60 potassium lactobionate, 20 taurine, 10 KH_2_PO_4_, 20 HEPES, 110 sucrose, and 1 g/L fatty acid BSA-free (pH 7.1) (MiR05; Oroboros Instruments, Innsbruck, Austria).

Freshly isolated quadriceps mitochondria (0.1 mg of mitochondrial protein per 2 mL chamber) were used for respiration measurement. For the assessment of mitochondrial respiration, the following protocol was used:

(1) non-phosphorylating LEAK respiration was assessed by injecting 10 mM Pyruvic acid sodium salt 10 mM L-glutamic acid and 2 mM L- malic acid as NADH (N)-linked substrates: state NL;
(2) OXPHOS capacity was induced by adding 1.25 mM ADP at saturating concentration: state NP;
(3) NADH and succinate (NS)-linked OXPHOS capacity was measured by adding 10 mM succinic acid: state NSP.

All acids were neutralized by KOH. 

The OXPHOS coupling efficiency (OCE) was calculated as measure of mitochondrial quality and control. OCE, calculated with the formula (1 − (state NL)/(state NP)), reflects the coupling of respiration supported by electron transferring flavoprotein (ETF) with pyruvate, glutamate, and malate as substrates before (state NL) and after addition of ADP (state NP). The OCE is noted between 0 and 1, while the RCR could be from 0 to infinite. A lower value of OCE means lesser coupling of the oxidation and phosphorylation after the addition of ADP. As the OCE decreases, it is, therefore, less coupled [61].

### 4.12. Statistical Analysis

Statistical analyses were performed using a software package Statistica v. 13.0 (StatSoft Inc., Tulsa, OK, USA). The results are expressed as the mean ± standard error (SEM). The differences in body mass were tested using mix model ANOVA. The differences between the means in other analyzed data were tested using one-way ANOVA. If a difference was detected in the ANOVA model, the significant differences were determined using Tukey’s post-hoc test. The results were considered statistically significant when *p* < 0.05. 

## 5. Conclusions

The present findings indicated that the nine days Dex treatment induces changes in blood lipid metabolites and modifies the long-term swimming-induced metabolic response in rats seen both in blood and skeletal muscle. It is worth emphasizing that these changes are not accompanied by damage to rats’ skeletal muscles or liver. Interestingly, prolonged swimming induced a different response in the levels of the metabolites mentioned above, especially in the white, fast-twitched EDL muscle. Despite these changes, there was no difference in aerobic and anaerobic enzymatic activities. This study shows for the first time the cumulative effect of exercise and Dex on selected elements of lipid metabolism, which seems to be essential for the patient’s health due to the common use of glucocorticoids like Dex.

## Figures and Tables

**Table 1:** The effects of Dex treatment, swimming, and a combination of these factors on liver and muscle damage markers and stress response elements.

	SC	SE	DC	DE	*p*
**CK** (U/L)	1125.47 ± 93.94	1310.71 ± 78.38	1365.28 ± 65.01	1263.82 ± 44.25	ns
**ALT** (U/L)	48.03 ± 1.32	44.42 ± 1.03	49.53 ± 1.78	49.35 ± 2.04	ns
**Cort** (pg/mL)	4.72 ± 0.84	5.47 ± 0.49 *	2.38 ± 0.35 *	3.46 ± 0.69	0.009 between *
**Adr** (pg/mL)	246.82 ± 22.03	186.98 ± 28.37	130.36 ± 19.24 *	109.40 ± 12.19 *	<0.05 between SC and *

Creatine kinase (CK), Alanine Aminotransferase (ALT) activities, and Cortisol (Cort) and Adrenaline (Adr) concentrations were measured in rats’ plasma in saline-treated, non-exercised (SC), saline-treated, exercised (SE), Dex-treated, non-exercised (DC) and Dex-treated, exercised (DE) groups of rats. The data are presented as the means ± SEM (*n* = 10 in each group for all parameters except Cort, *n* = 7). Meanning of * is explain in the *p* part of table.

**Table 2:** The effects of Dex treatment, swimming, and a combination of these factors on activities of enzymes related to aerobic and anaerobic metabolism in skeletal muscles.

	*Soleus*					*Extensor Digitorum Longus*				
Enzyme	SC	SE	DC	DE	*p*	SC	SE	DC	DE	*p*
**CS** (nmol/min/mg of protein)	60.11 ± 1.68	56.89 ± 0.92	59.25 ± 1.95	54.53 ± 1.26	ns	43.53 ± 1.03	40.08 ± 1.30	42.09 ± 1.67	44.47 ± 1.36	ns
**COX** (nmol/min/mg of protein)	62.03 ± 1.57	59.59 ± 1.81	63.68 ± 1.42	63.82 ± 1.72	ns	44.83 ± 1.39	47.04 ± 1.39	42.70 ± 1.67	45.63 ± 1.54	ns
**LDH 0.3** (μmol/min/mg of protein)	1.02 ± 0.03	0.99 ± 0.03	1.05 ± 0.03	1.01 ± 0.03	ns	2.83 ± 0.09	2.95 ± 0.10	2.97 ± 0.09	2.92 ± 0.08	ns
**LDH 2.1** (μmol/min/mg of protein)	0.60 ± 0.01	0.60 ± 0.02	0.64 ± 0.02	0.58 ± 0.01	ns	2.66 ± 0.09	2.83 ± 0.06	2.90 ± 0.09	2.75 ± 0.05	ns

Citrate synthase (CS), cytochrome c oxidase (COX), lactate dehydrogenases (LDH) 0.3 and 2.1 activities were measured in rats’ soleus and extensor digitorum longus homogenates in saline-treated, non-exercised (SC), saline-treated, exercised (SE), Dex-treated, non-exercised (DC) and Dex-treated, exercised (DE) groups of rats. The data are presented as the means ± SEM (*n* = 10 in each group).

**Table 3:** The effects of Dex treatment, swimming, and a combination of these factors on skeletal muscle mitochondrial bioenergetics.

	SC	SE	DC	DE	*p*
**state NL**	20.01 ± 1.18	17.79 ± 2.18	20.67 ± 1.45	20.12 ± 1.58	ns
**state NP**	377.77 ± 31.21	344.07 ± 34.84	334.21 ± 30.43	375.47 ± 41.54	ns
**state NSP**	561.77 ± 31.21	521.86 ± 44.24	570.21 ± 37.40	512.27 ± 27.51	ns
**OCE**	0.92 ± 0.012	0.93 ± 0.005	0.93 ± 0.003	0.94 ± 0.005	ns

Non-phosphorylating LEAK respiration (state NL), OXPHOS capacity (state NP), NADH and succinate (NS)-linked OXPHOS capacity (state NSP), and OXPHOS coupling efficiency (OCE) were measured in quadriceps mitochondria in saline-treated, non-exercised (SC), saline-treated, exercised (SE), Dex-treated, non-exercised (DC) and Dex-treated, exercised (DE) groups of rats. The data are presented as the means ± SEM (*n* = 9 in each group).

## Data Availability

The data presented in this study are available on request from the corresponding author.

## Supplementary Materials

The following supporting information can be downloaded at: https://www.mdpi.com/article/10.3390/ijms23020748/s1.

## References

[1] Van Staa T.P., Leufkens H.G., Abenhaim L., Begaud B., Zhang B., Cooper C. (2000). Use of oral corticosteroids in the United Kingdom. QJM Mon. J. Assoc. Physicians.

[2] Benard-Laribiere A., Pariente A., Pambrun E., Begaud B., Fardet L., Noize P. (2017). Prevalence and prescription patterns of oral glucocorticoids in adults: A retrospective cross-sectional and cohort analysis in France. BMJ Open.

[3] Waljee A.K., Rogers M.A., Lin P., Singal A.G., Stein J.D., Marks R.M., Ayanian J.Z., Nallamothu B.K. (2017). Short term use of oral corticosteroids and related harms among adults in the United States: Population based cohort study. BMJ.

[4] Kroot E.J., Huisman A.M., Van Zeben J., Wouters J.M., Van Paassen H.C. (2006). Oral pulsed dexamethasone therapy in early rheumatoid arthritis: A pilot study. Ann. N. Y. Acad. Sci..

[5] Okano M. (2009). Mechanisms and clinical implications of glucocorticosteroids in the treatment of allergic rhinitis. Clin. Exp. Immunol..

[6] Tashkin D.P., Strange C. (2018). Inhaled corticosteroids for chronic obstructive pulmonary disease: What is their role in therapy?. Int. J. Chronic Obstr. Pulm. Dis..

[7] Pufall M.A. (2015). Glucocorticoids and Cancer. Adv. Exp. Med. Biol..

[8] Malkawi A.K., Masood A., Shinwari Z., Jacob M., Benabdelkamel H., Matic G., Almuhanna F., Dasouki M., Alaiya A.A., Rahman A.M.A. (2019). Proteomic Analysis of Morphologically Changed Tissues after Prolonged Dexamethasone Treatment. Int. J. Mol. Sci..

[9] Nguelefack-Mbuyo E.P., Peyembouo F.P., Fofie C.K., Nguelefack T.B. (2021). Dose-dependent and time-dependent metabolic, hemodynamic, and redox disturbances in dexamethasone-treated Wistar rats. J. Basic Clin. Physiol. Pharmacol..

[10] De Vos P., Saladin R., Auwerx J., Staels B. (1995). Induction of ob gene expression by corticosteroids is accompanied by body weight loss and reduced food intake. J. Biol. Chem..

[11] Son Y.H., Lee S.J., Lee K.B., Lee J.H., Jeong E.M., Chung S.G., Park S.C., Kim I.G. (2015). Dexamethasone downregulates caveolin-1 causing muscle atrophy via inhibited insulin signaling. J. Endocrinol..

[12] Chang J.S., Kong I.D. (2020). Irisin prevents dexamethasone-induced atrophy in C2C12 myotubes. Pflug. Arch. Eur. J. Physiol..

[13] Qin J., Du R., Yang Y.Q., Zhang H.Q., Li Q., Liu L., Guan H., Hou J., An X.R. (2013). Dexamethasone-induced skeletal muscle atrophy was associated with upregulation of myostatin promoter activity. Res. Vet. Sci..

[14] Liu J., Peng Y., Wang X., Fan Y., Qin C., Shi L., Tang Y., Cao K., Li H., Long J. (2016). Mitochondrial Dysfunction Launches Dexamethasone-Induced Skeletal Muscle Atrophy via AMPK/FOXO3 Signaling. Mol. Pharm..

[15] Dahabiyeh L.A., Malkawi A.K., Wang X., Colak D., Mujamammi A.H., Sabi E.M., Li L., Dasouki M., Abdel Rahman A.M. (2020). Dexamethasone-Induced Perturbations in Tissue Metabolomics Revealed by Chemical Isotope Labeling LC-MS analysis. Metabolites.

[16] Poggioli R., Ueta C.B., Drigo R.A., Castillo M., Fonseca T.L., Bianco A.C. (2013). Dexamethasone reduces energy expenditure and increases susceptibility to diet-induced obesity in mice. Obesity.

[17] Dumas J.F., Simard G., Roussel D., Douay O., Foussard F., Malthiery Y., Ritz P. (2003). Mitochondrial energy metabolism in a model of undernutrition induced by dexamethasone. Br. J. Nutr..

[18] Koski C.L., Rifenberick D.H., Max S.R. (1974). Oxidative metabolism of skeletal muscle in steroid atrophy. Arch. Neurol..

[19] Weber K., Bruck P., Mikes Z., Kupper J.H., Klingenspor M., Wiesner R.J. (2002). Glucocorticoid hormone stimulates mitochondrial biogenesis specifically in skeletal muscle. Endocrinology.

[20] Momken I., Stevens L., Bergouignan A., Desplanches D., Rudwill F., Chery I., Zahariev A., Zahn S., Stein T.P., Sebedio J.L. (2011). Resveratrol prevents the wasting disorders of mechanical unloading by acting as a physical exercise mimetic in the rat. FASEB J. Off. Publ. Fed. Am. Soc. Exp. Biol..

[21] Alamdari N., Aversa Z., Castillero E., Gurav A., Petkova V., Tizio S., Hasselgren P.O. (2012). Resveratrol prevents dexamethasone-induced expression of the muscle atrophy-related ubiquitin ligases atrogin-1 and MuRF1 in cultured myotubes through a SIRT1-dependent mechanism. Biochem. Biophys. Res. Commun..

[22] Liu C.W., Huang C.C., Hsu C.F., Li T.H., Tsai Y.L., Lin M.W., Tsai H.C., Huang S.F., Yang Y.Y., Hsieh Y.C. (2020). SIRT1-dependent mechanisms and effects of resveratrol for amelioration of muscle wasting in NASH mice. BMJ Open Gastroenterol..

[23] Huang Y., Zhu X., Chen K., Lang H., Zhang Y., Hou P., Ran L., Zhou M., Zheng J., Yi L. (2019). Resveratrol prevents sarcopenic obesity by reversing mitochondrial dysfunction and oxidative stress via the PKA/LKB1/AMPK pathway. Aging.

[24] Macedo A.G., Krug A.L., Herrera N.A., Zago A.S., Rush J.W., Amaral S.L. (2014). Low-intensity resistance training attenuates dexamethasone-induced atrophy in the flexor hallucis longus muscle. J. Steroid Biochem. Mol. Biol..

[25] Krug A.L., Macedo A.G., Zago A.S., Rush J.W., Santos C.F., Amaral S.L. (2016). High-intensity resistance training attenuates dexamethasone-induced muscle atrophy. Muscle Nerve.

[26] Barel M., Perez O.A., Giozzet V.A., Rafacho A., Bosqueiro J.R., do Amaral S.L. (2010). Exercise training prevents hyperinsulinemia, muscular glycogen loss and muscle atrophy induced by dexamethasone treatment. Eur. J. Appl. Physiol..

[27] Sato K., Nishiguchi K.M., Maruyama K., Moritoh S., Fujita K., Ikuta Y., Kasai H., Nakazawa T. (2016). Topical ocular dexamethasone decreases intraocular pressure and body weight in rats. J. Negat. Results Biomed..

[28] Noh K.K., Chung K.W., Choi Y.J., Park M.H., Jang E.J., Park C.H., Yoon C., Kim N.D., Kim M.K., Chung H.Y. (2014). beta-Hydroxy beta-methylbutyrate improves dexamethasone-induced muscle atrophy by modulating the muscle degradation pathway in SD rat. PLoS ONE.

[29] Minetto M.A., Botter A., Lanfranco F., Baldi M., Ghigo E., Arvat E. (2010). Muscle fiber conduction slowing and decreased levels of circulating muscle proteins after short-term dexamethasone administration in healthy subjects. J. Clin. Endocrinol. Metab..

[30] Hedya S., Hawila N., Abdin A., Abd Elmaaboud M. (2019). Luteolin Attenuates Dexamethasone-Induced Skeletal Muscle Atrophy in Male Albino Rats. Med. J. Cairo Univ..

[31] Jackson E.R., Kilroy C., Joslin D.L., Schomaker S.J., Pruimboom-Brees I., Amacher D.E. (2008). The early effects of short-term dexamethasone administration on hepatic and serum alanine aminotransferase in the rat. Drug Chem. Toxicol..

[32] Fang M., Zhang Q., Yu P., Ge C., Guo J., Zhang Y., Wang H. (2020). The effects, underlying mechanism and interactions of dexamethasone exposure during pregnancy on maternal bile acid metabolism. Toxicol. Lett..

[33] Loose D.S., Do Y.S., Chen T.L., Feldman D. (1980). Demonstration of glucocorticoid receptors in the adrenal cortex: Evidence for a direct dexamethasone suppressive effect on the rat adrenal gland. Endocrinology.

[34] Pasquali R., Ambrosi B., Armanini D., Cavagnini F., Uberti E.D., Del Rio G., de Pergola G., Maccario M., Mantero F., Marugo M. (2002). Cortisol and ACTH response to oral dexamethasone in obesity and effects of sex, body fat distribution, and dexamethasone concentrations: A dose-response study. J. Clin. Endocrinol. Metab..

[35] Ebata T., Hayasaka H. (1979). Effects of aldosterone and dexamethasone on blood chemical mediators in endotoxin shock. Jpn. J. Surg..

[36] Richter E.A., Hargreaves M. (2013). Exercise, GLUT4, and skeletal muscle glucose uptake. Physiol. Rev..

[37] Huertas J.R., Casuso R.A., Agustin P.H., Cogliati S. (2019). Stay Fit, Stay Young: Mitochondria in Movement: The Role of Exercise in the New Mitochondrial Paradigm. Oxidative Med. Cell. Longev..

[38] Buren J., Lai Y.C., Lundgren M., Eriksson J.W., Jensen J. (2008). Insulin action and signalling in fat and muscle from dexamethasone-treated rats. Arch. Biochem. Biophys..

[39] Dumas J.F., Bielicki G., Renou J.P., Roussel D., Ducluzeau P.H., Malthiery Y., Simard G., Ritz P. (2005). Dexamethasone impairs muscle energetics, studied by (31)P NMR, in rats. Diabetologia.

[40] Brooks G.A. (2020). The tortuous path of lactate shuttle discovery: From cinders and boards to the lab and ICU. J. Sport Health Sci..

[41] Sun J., Ye X., Xie M., Ye J. (2016). Induction of triglyceride accumulation and mitochondrial maintenance in muscle cells by lactate. Sci. Rep..

[42] Romijn J.A., Coyle E.F., Sidossis L.S., Gastaldelli A., Horowitz J.F., Endert E., Wolfe R.R. (1993). Regulation of endogenous fat and carbohydrate metabolism in relation to exercise intensity and duration. Am. J. Physiol..

[43] Muscella A., Stefano E., Lunetti P., Capobianco L., Marsigliante S. (2020). The Regulation of Fat Metabolism During Aerobic Exercise. Biomolecules.

[44] Xu C., He J., Jiang H., Zu L., Zhai W., Pu S., Xu G. (2009). Direct effect of glucocorticoids on lipolysis in adipocytes. Mol. Endocrinol..

[45] Macdonald T.L., Wan Z., Frendo-Cumbo S., Dyck D.J., Wright D.C. (2013). IL-6 and epinephrine have divergent fiber type effects on intramuscular lipolysis. J. Appl. Physiol..

[46] Schippers M.-P., Ramirez O., Arana M., McClelland G.B. (2021). Increased Reliance on Carbohydrates for Aerobic Exercise in Highland Andean Leaf-Eared Mice, but Not in Highland Lima Leaf-Eared Mice. Metabolites.

[47] Liang H., Ward W.F. (2006). PGC-1alpha: A key regulator of energy metabolism. Adv. Physiol. Educ..

[48] Mitsui T., Azuma H., Nagasawa M., Iuchi T., Akaike M., Odomi M., Matsumoto T. (2002). Chronic corticosteroid administration causes mitochondrial dysfunction in skeletal muscle. J. Neurol..

[49] Pawlikowska P., Lokociejewska M., Pajak B., Gajewska M., Jank M., Hocquette J.-F., Orzechowski A. (2008). Metabolic programming establishes resistance of oxidative-type skeletal muscles to glucocorticoid-induced muscle cachexia in rats. Arch. Für Tierz..

[50] Chen S., Zhou L., Sun J., Qu Y., Chen M. (2021). The Role of cAMP-PKA Pathway in Lactate-Induced Intramuscular Triglyceride Accumulation and Mitochondria Content Increase in Mice. Front. Physiol..

[51] Wang J.C., Gray N.E., Kuo T., Harris C.A. (2012). Regulation of triglyceride metabolism by glucocorticoid receptor. Cell Biosci..

[52] Amin S.B., Sinkin R.A., McDermott M.P., Kendig J.W. (1999). Lipid intolerance in neonates receiving dexamethasone for bronchopulmonary dysplasia. Arch. Pediatrics Adolesc. Med..

[53] Flis D.J., Olek R.A., Kaczor J.J., Rodziewicz E., Halon M., Antosiewicz J., Wozniak M., Gabbianelli R., Ziolkowski W. (2016). Exercise-Induced Changes in Caveolin-1, Depletion of Mitochondrial Cholesterol, and the Inhibition of Mitochondrial Swelling in Rat Skeletal Muscle but Not in the Liver. Oxidative Med. Cell. Longev..

[54] Maughan R.J. (1982). A simple, rapid method for the determination of glucose, lactate, pyruvate, alanine, 3-hydroxybutyrate and acetoacetate on a single 20-mul blood sample. Clin. Chim. Acta Int. J. Clin. Chem..

[55] De Lisio M., Kaczor J.J., Phan N., Tarnopolsky M.A., Boreham D.R., Parise G. (2011). Exercise training enhances the skeletal muscle response to radiation-induced oxidative stress. Muscle Nerve.

[56] Wharton D.C., Tzagoloff A., Estabrook R.W., John A. (1967). Cytochrome C oxidase from beef heart mitochondria. Methods Enzymol.

[57] Leger L.A., Taylor A.W. (1982). The chronic effects of continuous and intermittent running upon lactate dehydrogenase activity of heart, fast and slow twitch muscles in the rat. J. Physiol..

[58] Halon-Golabek M., Borkowska A., Kaczor J.J., Ziolkowski W., Flis D.J., Knap N., Kasperuk K., Antosiewicz J. (2018). hmSOD1 gene mutation-induced disturbance in iron metabolism is mediated by impairment of Akt signalling pathway. J. Cachexia Sarcopenia Muscle.

[59] Cieminski K., Flis D.J., Dzik K., Kaczor J.J., Czyrko E., Halon-Golabek M., Wieckowski M.R., Antosiewicz J., Ziolkowski W. (2021). Swim training affects Akt signaling and ameliorates loss of skeletal muscle mass in a mouse model of amyotrophic lateral sclerosis. Sci. Rep..

[60] Fontaine E., Eriksson O., Ichas F., Bernardi P. (1998). Regulation of the permeability transition pore in skeletal muscle mitochondria. Modulation By electron flow through the respiratory chain complex i. J. Biol. Chem..

[61] van Schaardenburgh M., Wohlwend M., Rognmo O., Mattsson E.J.R. (2017). Exercise in claudicants increase or decrease walking ability and the response relates to mitochondrial function. J. Transl. Med..

