# Correction: Flis et al. The Effect of Long-Lasting Swimming on Rats Skeletal Muscles Energy Metabolism after Nine Days of Dexamethasone Treatment. *Int. J. Mol. Sci.* 2022, *23*, 748

**DOI:** 10.3390/ijms26052165

**Published:** 2025-02-28

**Authors:** Damian Jozef Flis, Emilia Gabriela Bialobrodzka, Ewa Aleksandra Rodziewicz-Flis, Zbigniew Jost, Andzelika Borkowska, Wieslaw Ziolkowski, Jan Jacek Kaczor

**Affiliations:** 1 Department of Pharmaceutical Pathophysiology, Faculty of Pharmacy, Medical University of Gdansk, Dębinki 7 Street, 80-211 Gdansk, Poland; 2 Poznan University of Physical Education, Królowej Jadwigi 27/39 Street, 61-871 Poznan, Poland; czyrko@awf.poznan.pl; 3 Department of Basic Physiotherapy, Gdansk University of Physical Education and Sport, K. Gorkiego 1 Street, 80-336 Gdansk, Poland; ewa.rodziewicz@awf.gda.pl; 4 Department of Biochemistry, Gdansk University of Physical Education and Sport, K. Gorkiego 1 Street, 80-336 Gdansk, Poland; zbigniew.jost@awf.gda.pl; 5 Department of Bioenergetics and Physiology of Exercise, Faculty of Health Sciences, Medical University of Gdansk, Dębinki 1 Street, 80-211 Gdansk, Poland; andzelika.borkowska@gumed.edu.pl; 6 Department of Rehabilitation Medicine, Faculty of Health Sciences, Medical University of Gdansk, Al. Zwycięstwa 30, 80-219 Gdansk, Poland; wieslaw.ziolkowski@gumed.edu.pl; 7 Department of Animal and Human Physiology, University of Gdansk, J. Bazynskiego 8 Street, 80-308 Gdansk, Poland; jan.kaczor@ug.edu.pl

Due to the lack of enzyme 17-α hydroxylase, rats and mice cannot produce appreciable cortisol; however, low cortisol concentrations are detectable in these rodents’ blood. After a discussion with the Academic Editor, we decide to remove data and details about cortisol from the manuscript [1] to avoid misleading readers about cortisol production by the rat.

**Table Legend**

In the original publication, there were data and descriptions related to cortisol in the legend for Table 1: The effects of Dex treatment, swimming, and a combination of these factors on liver and muscle damage markers and stress response elements.

**Error in Table**

In the original publication, there was information about cortisol in Table 1: The effects of Dex treatment, swimming, and a combination of these factors on liver and muscle damage markers and stress response elements, as published. 

The corrected Table 1 appears below. 

**Text Correction**

There was information about cortisol in the original publication.

A correction has been made to the Results section:

## 2.2. Effects of Dex Treatment and Exercise on Liver and Muscle Damage Markers and Stress Response Elements

Neither nine days of Dex treatment and three hours of swimming nor a combination of these conditions resulted in a change in blood creatine kinase (CK) and alanine aminotransferase (ALT) activities. However, there was a trend toward increasing CK activity in the rats’ blood after Dex administration (*p* = 0.1). Dex treatment also resulted in a lowered plasma adrenaline (Adr) concentration compared to the SC group. There was also a slight, insignificant decrease in the Adr concentration after swimming in both the SE and DE groups compared to the SC and DC groups (Table 1).

The Discussion section, paragraph 2:

One of the most common adverse events after Dex treatment is lowering body mass, which was also observed in our study. Interestingly, even topical ocular Dex treatment induced body mass loss, liver damage, and blood cholesterol alteration in rats [27]. Therefore, to evaluate whether the intraperitoneal injection of Dex, swimming, and combining these factors induce muscle or liver damage or influence stress response elements, we measured CK, ALT activities, and Adr concentrations in the rats’ plasma. As mentioned before, we observed changes in neither skeletal muscle nor liver damage markers, but we observed a tendency to increase the plasma CK activity after Dex treatment. This observation is in line with previously published papers, because, on the one hand, Noh and coworkers documented that 5 days of Dex administration resulted in a threefold increase in the CK serum level in rats [28]. However, on the other hand, other authors showed that Dex administration might even decrease the level of circulating CK levels, which may be related to protein synthesis inhibition, cellular membrane stabilization, anti-inflammatory effects, and catabolic effects on myofibers [29,30]. Changes in the level of blood ALT are Dex dose-dependent. In higher-than-clinical-dose levels of Dex treatment, the ALT increased by almost 10-fold after 4 days of administration [31], but the lower dosage of Dex did not alter blood ALT activity even after 12 days [32]. To evaluate whether Dex treatment or swimming acts as a stressor for animals, we measured the Adr concentration in rats’ plasma. After Dex treatment, the Adr level drops, which may be related to Dex’s inhibitory action on releasing this hormone [33]. No changes in the Adr level were observed after exercise in both groups.

The Materials and Methods section:

We removed Section 4.7: Blood Cortisol Concentration

The cortisol concentration was measured in plasma using an Arbor Assays Cortisol enzyme immunoassay kit (cat. number K003-H1/H5 Arbor Assays, Ann Arbor, MI, USA), according to the manufacturer’s instructions.

As the above content is modified, the reference number and section number are also modified accordingly. The authors apologize for any inconvenience caused and state that the scientific conclusions are unaffected. This correction was approved by the Academic Editor. The original publication has also been updated.

## Figures and Tables

**Table 1:** The effects of Dex treatment, swimming, and a combination of these factors on liver and muscle damage markers and stress response elements.

	SC	SE	DC	DE	*p*
**CK** (U/L)	1125.47 ± 93.94	1310.71 ± 78.38	1365.28 ± 65.01	1263.82 ± 44.25	ns
**ALT** (U/L)	48.03 ± 1.32	44.42 ± 1.03	49.53 ± 1.78	49.35 ± 2.04	ns
**Adr** (pg/mL)	246.82 ± 22.03	186.98 ± 28.37	130.36 ± 19.24 *	109.40 ± 12.19 *	<0.05 between SC and *

Creatine kinase (CK), Alanine Aminotransferase (ALT) activities, and Adrenaline (Adr) concentration were measured in rats’ plasma in saline-treated, non-exercised (SC), saline-treated, exercised (SE), Dex-treated, non-exercised (DC), and Dex-treated, exercised (DE) groups of rats. The data are presented as the means ± SEM (*n* = 10 in each group). Meaning of * is explain in the p part of table, ns—non significant.
