# Supplementary material for: The Effect of Long-Lasting Swimming on Rats Skeletal Muscles Energy Metabolism after Nine Days of Dexamethasone Treatment
Source: Int J Mol Sci. 2022 Jan 11;23(2):748. doi: 10.3390/ijms23020748 (PMC8775511; doi:10.3390/ijms23020748)
Supplement: Supplementary file 1 [file ijms-23-00748-s001.zip › ijms-1489944-supplementary.pdf]

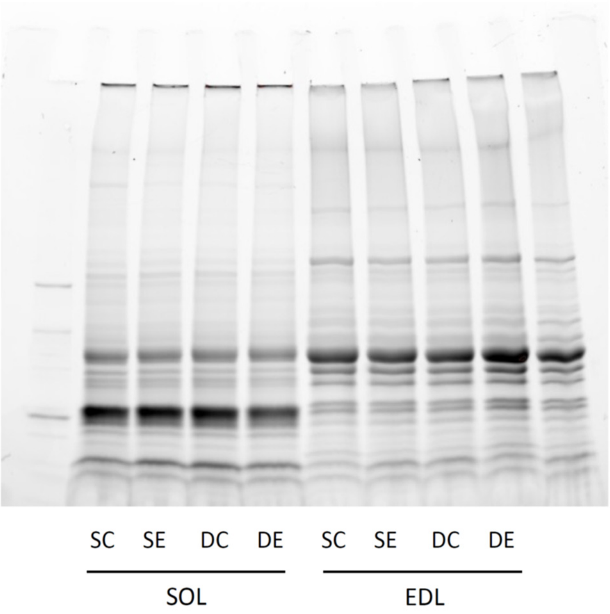

Figure S1. Sample picture with the total amount of protein on the membrane.

Changes in protein levels were assessed by densitometry of the immunoreactive bands and normalized to the total amount of protein in the samples transferred onto the membrane.
